# Implementation of specialised care improves survival in patients with amyotrophic lateral sclerosis

**DOI:** 10.1186/s42466-026-00528-x

**Published:** 2026-09-02

**Authors:** Omar Keritam, Daniel Bormann, Friedrich Haimberger, Diana Gharib, Merve Sener, Felix Gruber, Lukasz Antoniewicz, Ivan Fedak, Andreas Renner, Bernhard Fasching, Sabine Ullrich, Rafael Paternostro, Werner Dolak, Eva K Masel, Othmar Schuhfried, Jakob Rath, Gudrun Zulehner, Martin Krenn, Fritz Zimprich, Hakan Cetin

**Affiliations:** 1 Department of Neurology, Medical University of Vienna, Waehringer Guertel 18-20, Vienna, 1090 Austria; 2 Comprehensive Center for Clinical Neurosciences and Mental Health, Medical University of Vienna, Vienna, Austria; 3 Austrian Federation of Social Insurances, Vienna, Austria; 4 Division of Pulmonology, Department of Medicine II, Medical University of Vienna, Vienna, Austria; 5 Comprehensive Center for Chest Diseases, Medical University of Vienna, Vienna, Austria; 6 Division of Gastroenterology and Hepatology, Department of Medicine III, Medical University of Vienna, Vienna, Austria; 7 Division of Palliative Medicine, Department of Medicine I, Medical University of Vienna, Vienna, Austria; 8 Department of Physical Medicine, Rehabilitation and Occupational Medicine, Medical University of Vienna, Vienna, Austria

**Keywords:** Motor neuron disease, Amyotrophic lateral sclerosis, ALS, Multidisciplinary care

## Abstract

**Objective:**

Access to multidisciplinary care influences survival in patients with amyotrophic lateral sclerosis (pwALS). However, real-world data comparing structured specialised care with general neurological management remain limited. We aimed to assess the influence of implementing a specialised outpatient clinic on survival in pwALS.

**Methods:**

This retrospective cohort study included pwALS meeting the Gold Coast criteria who were treated at the Department of Neurology of the Medical University of Vienna between January 2009 and July 2023. Demographic and clinical parameters and survival data were obtained from the local ALS registry, the *Austrian Federation of Social Insurance* databases, and the national mortality database of *Statistik Austria*. Data were censored in December 2024. Outcomes were compared between patients managed before (general care cohort) and after the establishment of a specialised ALS outpatient clinic in 2018 (specialised care cohort).

**Results:**

A total of 242 pwALS were included (47.5% female), of whom 43.8% received general neurological care and 56.2% specialised care. Spinal onset ALS was observed in 65.1% and 70.6%, respectively. Baseline demographic and clinical characteristics were comparable between both cohorts. Median survival time was 27.0 months (95% CI 23.0–35.0) in the general care cohort and 40.0 months (95% CI 32.0–47.0) in specialised care cohort (*p* = 0.0173). This survival difference was driven by patients with spinal onset ALS, whereas no benefit was observed in those with bulbar onset disease.

**Conclusions:**

Specialised care was associated with improved survival in this real-world ALS cohort, likely reflecting the cumulative effect of coordinated multidisciplinary management rather than individual interventions.

**Supplementary Information:**

The online version contains supplementary material available at https://doi.org/10.1186/s42466-026-00528-x.

## Introduction

The clinical spectrum of amyotrophic lateral sclerosis (ALS) is characterised by substantial inter-individual variability in site of symptom onset, rate and pattern of disease progression, and relative involvement of upper and lower motor neurons. These factors are associated with distinct prognostic trajectories and survival across clinical phenotypes [1–7], highlighting the importance of phenotypic classification in ALS [8]. Beyond these biological differences, disparities in healthcare infrastructure and access to multidisciplinary care including rehabilitation and palliative care services, respiratory support, and nutritional management may further influence quality of life and survival in ALS. Notably, multidisciplinary care of ALS patients in particular has been associated with improved outcome, but robust real-world data comparing general management with structured specialised care pathways remain limited [9–14].

The establishment of a specialised ALS outpatient clinic at the Department of Neurology of the Medical University of Vienna in 2018 provided a valuable opportunity to assess the impact of this care model on survival by the comparison of outcomes before and after 2018. We further explored differences in healthcare utilisation and treatment patterns between general and specialised care settings to correlate outcome differences with specific aspects of multidisciplinary management.

## Methods

### Study design and patient ascertainment

In this retrospective cohort study, data were derived from a local registry comprising clinical and demographic information at the Department of Neurology of the Medical University of Vienna. The study covered the period from January 2009 to July 2023, capturing both the period before and after the establishment of the specialised ALS outpatient clinic in January 2018. Before 2018, patients diagnosed at the Department of Neurology of the Medical University of Vienna received general neurological care in non-specialised hospitals or office-based practices without structured multidisciplinary management. From January 2018 onwards, by contrast, all pwALS received continuous, coordinated multidisciplinary care. Clinical records were retrospectively reviewed to identify eligible pwALS who met the Gold Coast diagnostic criteria [15, 16]. Although these criteria include progressive muscular atrophy (PMA), this phenotype is typically associated with a more protracted disease course [5, 7]. Therefore, patients with PMA, together with those diagnosed with other motor neuron diseases, including primary lateral sclerosis, spinal muscular atrophy, and spinal and bulbar muscular atrophy, were excluded to ensure a prognostically more homogenous study population. The final study dataset was linked to the hospital discharge and prescription databases of the *Austrian Federation of Social Insurances*, covering the time period between January 2013 and December 2023, enabling analyses of healthcare utilisation and treatment patterns. Survival status was ascertained through linkage with national mortality data from *Statistik Austria*, with follow-up censored on December 31, 2024. The present cohort has been previously reported as part of a comprehensive analysis of the demographic, clinical and genetic characteristics of pwALS in Austria [5].

### Data collection

Clinical and demographic data were extracted from the local ALS registry and electronic health records. Collected parameters included sex, age at onset, region of onset, diagnostic delay, King’s stage at diagnosis (derived from the documented patient history and neurological examination), progression rate of the ALS Functional Rating Scale Revised (ALSFRS-R PR; calculated as [48–last ALSFRS-R score]/months from onset to last assessment), loss per month of the body mass index (BMI), and treatments (riluzole, invasive ventilation [IV], non-invasive ventilation [NIV], percutaneous endoscopic gastrostomy [PEG]). Anonymised data on post-diagnosis hospitalisations, rehabilitation therapies (physiotherapy, occupational therapy, speech therapy), and dispensed medications were obtained from the *Austrian Federation of Social Insurances*. Pharmaceutical data included all medications dispensed after diagnosis and were categorised according to the fourth level of the Anatomical Therapeutic Chemical (ATC) classification (https://atcddd.fhi.no/). Exposure to a specific medication in individual patients was only considered upon a minimum of two dispensations. To ensure clinically meaningful results, medications were included only when at least 10% of pwALS were exposed to them during the observational period.

### Statistical analysis

Descriptive analyses were performed using means with standard deviations (SD) or medians with interquartile ranges (IQR), as appropriate, and compared between management periods, i.e. general neurological care (GNC) before January 2018 and specialised multidisciplinary care (SMC) from January 2018 onwards using t-test, Mann-Whitney test or the Fisher’s exact test. Associations between medication exposure at the fourth ATC level and care model (GNC vs. SMC) were quantified using odds ratios (OR) with 95% confidence intervals (CI), based on conditional maximum likelihood estimates derived from Fisher’s exact test. Given the exploratory nature of these analyses, no adjustment for multiple comparisons was applied. Estimates were derived for the overall post-diagnosis period as well as stratified by time since diagnosis (i.e., years 1–3). Kaplan-Meier analyses with log-rank tests were used to compare IV-free survival from symptom onset between care models in the overall cohort as well as in the spinal onset and bulbar onset ALS subgroups. Cox proportional hazards regression was used to assess the association between care model and survival, including an interaction term to evaluate whether the effect of SMC depended on the region of onset (T ~ sex + age at onset + King’s stage at diagnosis + onset region + care model + onset region × care model). Hazard ratios (HR) with 95% CIs were reported. To explore the potential influence of diagnostic delay and assess whether the association between care model and survival varied over time, we performed a post-hoc, multi-layered sensitivity analyses using diagnosis rather than symptom onset as the time origin. Kaplan-Meier analyses were repeated using both the log-rank and Gehan-Breslow-Wilcoxon tests. In addition, Cox regression models anchored at diagnosis were fitted with follow-up censored at fixed horizons of 12, 24, and 36 months, including diagnostic delay as an additional covariate. Finally, a piecewise Cox regression model with time-splitting at 24 months was performed to confirm results from the fixed-horizon analyses. A two-sided p-value of < 0.05 was considered statistically significant. All analyses and graphical illustrations were performed using R version 4.4.1 (R Project for Statistical Computing), RStudio version 2024.09.0 + 375 (RStudio PBC) and GraphPad Prism version 10.6.0 for macOS (GraphPad Software, Boston MA, USA).

## Results

### Clinical and demographic characteristics

A total of 242 patients were included in this study (47.5% female), of whom 106 patients (43.8%) received GNC and 136 patients (56.2%) received SMC (Table 1). Patients with spinal onset ALS accounted for 65.1% of the GNC subgroup and 70.6% of the SMC subgroup (Supplementary Tables 1 and 2). Mean age at onset was 60.2 (SD 11.9) and 61.1 years (SD 11.8), respectively. King’s stage at diagnosis, median ALSFRS-R PR, median BMI loss, as well as the use of riluzole, PEG, NIV, and IV did not differ significantly between the general care and specialised care subgroups. Median diagnostic delay was significantly longer in the SMC subgroup (11.0 months [IQR 6.0–17.0] vs. 9.0 months [IQR 5.0–12.0], *p* = 0.0081).

**Table 1:** Demographic and clinical characteristics of patients receiving general neurological care and patients with specialised multidisciplinary care

	Whole cohort *n* = 242	GNC cohort *n* = 106	SMC cohort *n* = 136	*p*-value
Sex (%)				
Female	115 (47.5)	46 (43.4)	69 (50.7)	0.2996
Male	127 (52.5)	60 (56.6)	67 (49.3)	
Onset subtype (%)				
Spinal	165 (68.2)	69 (65.1)	96 (70.6)	0.4049
Bulbar (or respiratory*)	77 (31.8)	37 (34.9)	40 (29.4)	
Mean age at onset, years (SD)	60.7 (11.8)	60.2 (11.9)	61.1 (11.8)	0.5583
Median diagnostic delay, months (IQR)	10.0 (6.0; 15.0)	9.0 (5.0; 12.0)	11.0 (6.0; 17.0)	0.0081
King’s stage at diagnosis (%)				
Stage I	41 (16.9)	16 (15.1)	25 (18.4)	0.3809
Stage II	79 (32.6)	38 (35.8)	41 (30.1)	
Stage III	93 (38.4)	43 (40.6)	50 (36.8)	
Stage IV	29 (12.0)	9 (8.5)	20 (14.7)	
Median ALSFRS-R progression per month (IQR)	-0.8 (-1.4; -0.5)	-0.8 (-1.2; -0.5)	-0.9 (-1.4; -0.5)	0.7277
Median time onset to last ALSFRS-R (IQR)	20.0 (13.0; 31.5)	20.0 (14.0; 36.0)	20.0 (13.0; 31.0)	0.6958
Unknown (%)	100 (41.3)	88 (83.0)	12 (8.8)	
Median BMI loss per month (IQR)	-0.1 (-0.3; 0.0)	-0.1 (-0.3; 0.0)	-0.1 (-0.4; 0.0)	0.9448
Unknown (%)	126 (52.1)	68 (64.2)	58 (42.6)	
Therapies (%)				
Riluzole	189 (78.1)	94 (88.7)	113 (83.1)	0.2858
Unknown	13 (5.4)	4 (3.8)	7 (5.1)	
Percutaneous endoscopic gastrostomy	59 (24.4)	24 (22.6)	35 (25.7)	0.6519
Unknown	6 (2.5)	3 (2.8)	2 (1.5)	
Invasive ventilation	5 (2.1)	3 (2.8)	2 (1.5)	0.6527
Unknown	13 (5.4)	9 (8.5)	4 (2.9)	
Non-invasive ventilation	26 (10.7)	7 (6.6)	19 (14.0)	0.0970
Unknown	11 (4.5)	6 (5.7)	3 (2.2)	

*Only three patients had respiratory onset ALS and were added to the bulbar onset ALS subgroup

Statistical analyses were conducted using the Fisher’s exact test, the t-test or the Mann-Whitney test. ALS=Amyotrophic Lateral Sclerosis. ALSFRS-R: Amyotrophic Lateral Sclerosis Functional Rating Scale Revised. BMI: Body Mass Index. GNC: General Neurological Care. IQR: Interquartile Range. SD: Standard Deviation. SMC: Specialised Multidisciplinary Care

### Exploratory analysis of healthcare utilisation and medication patterns

Data from the hospital discharge and prescription databases were available for 62 patients in the GNC subgroup and 116 patients in the SMC subgroup in the respective post-diagnosis time period (Table 2). The median number of hospitalisations per year was significantly higher in the SMC subgroup (3.4 [IQR 1.1–7.2] vs. 1.2 [IQR 0.0-3.5], *p* < 0.0001), but the number of prescribed ATC level 4 medications per year were comparable in both groups, and the proportion of patients receiving physiotherapy, occupational therapy or speech therapy did not differ significantly between the two care settings. Exploratory analysis of drug dispensation patterns revealed no associations between specific medication groups and care setting (Supplementary Figs. 1–4).

**Table 2:** Additional parameters obtained from the database of the *Austrian Federation of Social Insurances*

	GNC cohort, *n* = 62	SMC cohort, *n* = 116	*p*-value
Median no. of hospitalisations per year (IQR)	1.2 (0.0–3.5)	3.4 (1.1–7.2)	< 0.0001
Median no. of prescribed medication groups per year (IQR)*	5.2 (2.7–10.4)	5.8 (2.9–11.1)	0.7590
Rehabilitation therapies (%)			
Physiotherapy	3 (4.8)	3 (2.6)	0.4211
Occupational therapy	4 (6.5)	9 (7.8)	1.0000
Speech therapy	7 (11.3)	16 (13.8)	0.8152

*by the 4th level of the Anatomical-Therapeutic-Chemical classification

Statistical analyses were conducted using the Fisher’s exact test or the Mann-Whitney test. GNC: General Neurological Care. IQR=Interquartile Range. SMC: Specialised Multidisciplinary Care

### Survival analysis

**Fig. 1:**
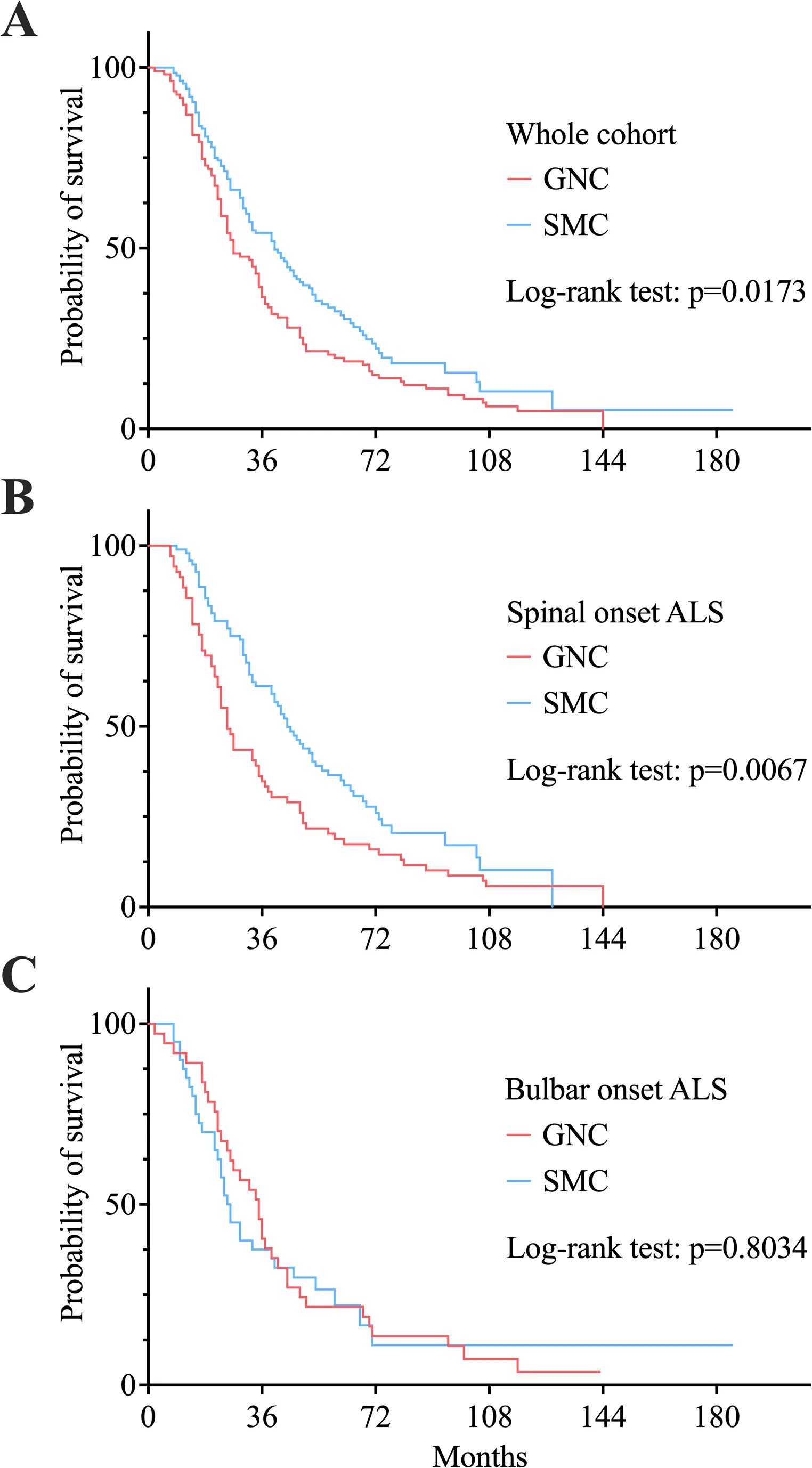
Kaplan-Meier analyses comparing IV-free survival from symptom onset between patients receiving GNC and patients receiving SMC in (**A**) the whole cohort (GNC 27.0 months [95% CI 23.0–35.0] vs. SMC 40.0 months [95% CI 32.0–47.0]), (**B**) the spinal onset ALS subgroup (GNC 25.0 months [95% CI 22.0–35.0] vs. SMC 44.0 months [95% CI 39.0–53.0]), and (**C**) the bulbar onset ALS subgroup (GNC 35.0 months [95% CI 25.0–41.0] vs. SMC 25.5 months [95% CI 21.0–39.0]). P-values were derived from the respective log-rank test, and median survival time with the corresponding 95% CI are reported. ALS: Amyotrophic Lateral Sclerosis. GNC: General Neurological Care. SMC: Specialised Multidisciplinary Care. 95% CI: 95% Confidence Interval

By the time of data censoring, 102 patients (96.2%) in the GNC group and 104 patients (76.5%) in the SMC group had died, while 3 (2.8%) and 2 (1.5%) had received IV, respectively. Median IV-free survival from symptom onset was 27.0 months (95% CI 23.0–35.0) in the GNC group and 40.0 months (95% CI 32.0–47.0) in the SMC group (*p* = 0.0173; Fig. 1A). Among patients with spinal onset ALS, median survival was markedly longer with specialised care (GNC: 25.0 months [95% CI 22.0–35.0] vs. SMC: 44.0 months [95% CI 39.0–53.0], *p* = 0.0067; Fig. 1B), but not in patients with bulbar onset ALS (Fig. 1C). These results were bolstered by the Cox regression analysis. Although neither care model nor onset region was independently associated with survival, a significant interaction between both variables was observed (Table 3). Specifically, patients with spinal-onset ALS receiving GNC had a significantly higher hazard than those receiving SMC (HR 2.13 [95% CI 1.16–3.91], *p* = 0.0156).

**Table 3:** Summary of the primary Cox regression model

Variable	HR estimate	95% CI	*p*-value
Sex [male]	1.50	1.11–2.02	0.0078
Age at onset	1.03	1.02–1.05	< 0.0001
King’s stage II at diagnosis	1.38	0.89–2.18	0.1586
King’s stage III at diagnosis	1.78	1.18–2.77	0.0057
King’s stage IV at diagnosis	1.54	0.89–2.63	0.1222
Onset region [spinal]	0.64	0.41–1.02	0.0583
Care model [GNC]	0.81	0.49–1.36	0.4256
Onset region [spinal] : Care model [GNC]	2.13	1.16–3.91	0.0156

GNC: General Neurological Care. HR: Hazard Ratio. 95% CI: 95% Confidence Interval

To explore the potential influence of diagnostic delay, we performed a post hoc sensitivity analysis using diagnosis rather than symptom onset as the time origin. Kaplan-Meier analyses showed no significant differences using the log-rank test. However, in the spinal-onset subgroup, the Gehan-Breslow-Wilcoxon test, which places greater weight on early events, demonstrated significantly longer survival in patients receiving SMC (29.0 months [95% CI 21.0–38.0]) than in those receiving GNC (16.5 months [95% CI 11.0–22.0], *p* = 0.0111; Supplementary Fig. 5). Consistent with this finding, Cox models censored at fixed follow-up horizons demonstrated a significant onset region × care model interaction at 12 and 24 months (HR 4.37 [95% CI 1.46–13.12], *p* = 0.0085, and HR 3.21 [95% CI 1.47–6.99], *p* = 0.0034, respectively), whereas this interaction was no longer significant at 36 months (Supplementary Tables 3–5). Likewise, the piecewise Cox model demonstrated that the effect of specialised multidisciplinary care differed significantly between the first 24 months after diagnosis and the subsequent follow-up period (three-way interaction HR 0.16 [95% CI 0.04–0.61], *p* = 0.0074). During the first 24 months after diagnosis, patients with spinal-onset ALS receiving GNC had a significantly higher hazard than those receiving SMC (HR 3.36 [95% CI 1.55–7.29], *p* = 0.0021), whereas no significant difference was observed thereafter (Supplementary Table 6). Diagnostic delay itself was not significantly associated with survival in any fixed-horizon or piecewise Cox model.

## Discussion

In this retrospective study, we analysed the impact of the implementation of SMC on survival of pwALS treated in an Austrian tertiary centre, leveraging social insurance discharge and prescription databases to provide insights into differences in healthcare utilisation and treatment patterns.

Our findings reveal a significant survival benefit associated with SMC compared with GNC, corresponding to an overall median difference of 13 months, while baseline characteristics were largely similar between both groups. These results are overall consistent with previous reports [9, 11–14, 17]. The survival benefit in our cohort was confined to patients with spinal onset disease, in whom the median difference was 19 months, whereas no significant benefit was observed in bulbar onset ALS patients. This divergence may reflect differences in disease course, as spinal onset ALS is typically associated with slower progression [4, 5, 18–21], potentially providing a longer therapeutic window during which specialised multidisciplinary management could have influenced disease course, whereas the more aggressive progression in bulbar onset disease may have limited the impact of specialised care. However, some studies have reported a more pronounced benefit in patients with bulbar onset ALS [14, 17], possibly reflecting earlier and more consistent initiation of interventions in those cohorts [12]. This raises the possibility that timely intervention within a narrow therapeutic window is particularly critical in bulbar onset ALS, given its generally shorter survival compared with limb onset ALS. Nevertheless, the reasons underlying these discrepancies across studies remain unclear.

One notable difference between the two cohorts was the significantly longer diagnostic delay in the SMC group. As diagnostic delay is an established surrogate of slower disease progression [22–24], we performed several complementary sensitivity analyses using diagnosis rather than symptom onset as the time origin. Kaplan-Meier analyses showed no significant difference in the log-rank test. However, the Gehan-Breslow-Wilcoxon test, which places greater weight on early events, demonstrated a significant survival benefit in patients with spinal-onset ALS receiving SMC. Likewise, fixed-horizon and piecewise Cox models consistently indicated that the survival benefit of SMC was most pronounced during the first 24 months after diagnosis, while diagnostic delay was not associated with survival. Together, these findings make it unlikely that the observed survival benefit is explained by the longer diagnostic delay in the SMC cohort and suggest that specialised multidisciplinary care exerts its greatest survival benefit early after diagnosis.

Earlier initiation of supportive measures including PEG and NIV has been shown to prolong survival in pwALS [25–27]. In our study, we did not identify substantial differences in the proportion of patients receiving key disease-modifying or supportive interventions, including riluzole, NIV, PEG and IV. Exploratory analyses of medication exposure also did not reveal consistent differences between care settings. However, patients receiving SMC were hospitalised more frequently after diagnosis than those receiving GNC, suggesting greater overall healthcare utilisation. This finding may reflect earlier recognition and management of complications or a lower threshold for elective admission for supportive interventions. Because the insurance records do not reliably capture the complications or procedures underlying individual admissions, these explanations cannot be distinguished in the present study. Taken together, these findings suggest that the survival benefit associated with specialised care is unlikely to be driven by single interventions alone but rather by the cumulative effect of coordinated multidisciplinary management, including structured follow-up, timely recognition of complications, and integrated decision-making across specialties, patients, caregivers, and treating physicians.

The SMC observation period coincided with a phase of considerable change in ALS care. Most notably, the guideline on motor neuron diseases, published by the German Society of Neurology in 2021 [28], reinforced recommendations for multidisciplinary management, including regular respiratory and nutritional monitoring and timely initiation of supportive interventions such as NIV and PEG. These developments may have further improved care during the latter part of the SMC observation period. Nevertheless, the introduction of specialised multidisciplinary care at our centre in 2018 represented the major structural change within our study period. Following its implementation, patients were reviewed at predefined 3-month intervals, extended to up to 6 months in those with slowly progressive disease, with the treating ALS neurologist acting as care coordinator. Regular clinical assessments guided timely referral to the relevant specialties, including pulmonology, gastroenterology, dietetics, speech therapy, physiotherapy, physical and rehabilitation medicine, and palliative care. Before 2018, by contrast, most patients discontinued follow-up at our centre shortly after diagnosis and were subsequently managed by office-based neurologists or in non-specialised hospitals, generally without structured multidisciplinary follow-up. This fragmented model of care may have delayed the recognition of complications and the initiation of supportive interventions. Overall, ALS should be regarded as a palliative disease throughout its course, with symptomatic treatment initiated early and adapted continuously to optimise symptom control and quality of life [29–32]. Looking ahead, the emergence of disease-modifying therapies such as tofersen will further increase the importance of specialised multidisciplinary care because of the need for genetic testing, intrathecal administration, and structured longitudinal monitoring.

Several limitations should be acknowledged. Diagnostic delay was significantly longer in the SMC cohort, which may, at least in part, reflect differences in referral pathways. Because diagnostic delay constitutes an additive component of survival from symptom onset, it could not be included as a covariate in the primary Cox model. We therefore performed several complementary post hoc sensitivity analyses using diagnosis rather than symptom onset as the time origin. Although these analyses consistently suggested that the observed survival benefit is unlikely to be explained by differences in diagnostic delay, residual confounding by unmeasured differences in disease progression cannot be completely excluded. Second, the retrospective study design precluded systematic assessment of several clinically relevant variables, including ALSFRS-R progression rate, monthly BMI loss, quality of life, the timing of supportive interventions, treatment intensity, adherence to guideline recommendations, and differences in clinical decision-making. Although King’s stage at diagnosis could be assigned to the entire cohort and was included in the adjusted analyses, ALSFRS-R progression rate and monthly BMI loss remained unavailable for a substantial proportion of patients, particularly those treated before establishment of the specialised multidisciplinary clinic. Third, calendar-time effects could not be fully accounted for, as pwALS were managed in different time periods (i.e., before and after 2018) during which standards of ALS care may have evolved. While such temporal improvements in ALS care may have differently influenced outcomes in both subgroups, this is unlikely to fully explain the observed survival difference, as the establishment of a specialised ALS outpatient clinic represents a distinct structural change in care delivery rather than a gradual temporal trend. Fourth, interventions delivered exclusively outside our centre without subsequent follow-up may not have been fully captured. Finally, the limited granularity of the nationwide social insurance database precluded detailed analysis of the observed differences in hospitalisations. The available records comprise discharge diagnoses only, whereas intercurrent complications and procedural codes are incompletely recorded, preventing reliable determination of the specific indications or interventions underlying individual admissions.

In conclusion, specialised multidisciplinary ALS care was associated with improved survival in this real-world cohort, particularly among patients with spinal onset disease. The integration of long-term clinical follow-up with nationwide hospital discharge and prescription data enabled a comprehensive assessment of healthcare utilisation and treatment patterns. Despite the absence of detectable differences in individual treatments, our findings support the implementation of structured multidisciplinary care pathways in ALS.

## Supplementary Information

Supplementary material 1.

## Data Availability

No patient data or study related documents are shared within this paper. Reasonable requests from qualified investigators will be considered by the corresponding author in accordance with applicable privacy regulations.
