# Supplementary material for: Implementation of specialised care improves survival in patients with amyotrophic lateral sclerosis
Source: Neurol Res Pract. 2026 Sep 2;8(1):73. doi: 10.1186/s42466-026-00528-x (PMC13540897; doi:10.1186/s42466-026-00528-x)
Supplement: Supplementary file 1 [file 42466_2026_528_MOESM1_ESM.docx]

**Supplementary appendix for the manuscript entitled**

**“Implementation of specialised care improves survival in patients with amyotrophic lateral sclerosis”**

Omar Keritam^1,2^, Daniel Bormann^1,2^, Friedrich Haimberger^1,2^, Diana Gharib^1,2^, Merve Sener^1,2^, Felix Gruber^3^, Lukasz Antoniewicz^4,5^, Ivan Fedak^4,5^, Andreas Renner^4,5^, Bernhard Fasching^1,2^, Sabine Ullrich^6^, Rafael Paternostro^6^, Werner Dolak^6^, Eva K Masel^7^, Othmar Schuhfried^8^, Jakob Rath^1,2^, Gudrun Zulehner^1,2^, Martin Krenn^1,2^, Fritz Zimprich^1,2^, Hakan Cetin^1,2^

^1^Department of Neurology, Medical University of Vienna, Vienna, Austria

^2^Comprehensive Center for Clinical Neurosciences and Mental Health, Medical University of Vienna, Vienna, Austria

^3^Austrian Federation of Social Insurances, Vienna, Austria

^4^Division of Pulmonology, Department of Internal Medicine II, Medical University of Vienna, Vienna, Austria

^5^Comprehensive Center for Chest Diseases, Medical University of Vienna, Vienna, Austria

^6^Division of Gastroenterology and Hepatology, Department of Internal Medicine III, Medical University of Vienna, Vienna, Austria

^7^Division of Palliative Medicine, Department of Internal Medicine I, Medical University of Vienna, Vienna, Austria

^8^Department of Physical Medicine, Rehabilitation and Occupational Medicine, Medical University of Vienna, Vienna, Austria

**Correspondence**

Ap. Prof. PD Dr. Hakan Cetin, DPhil

Department of Neurology

Medical University of Vienna

Waehringer Guertel 18-20, 1090 Vienna

Phone: +43 1 40400 31450

Fax: +43 1 40400 31410

hakan.cetin@meduniwien.ac.at

**Supplementary table 1** Demographic and clinical characteristics of patients with spinal onset amyotrophic lateral sclerosis, GNC cohort vs. SMC cohort.

|  | **Spinal onset, GNC**  **n=69** | **Spinal onset, SMC**  **n=96** | **p-value** |
| --- | --- | --- | --- |
| Sex (%) |  |  |  |
| Female | 30 (43.5) | 43 (44.8) | 0.8753 |
| Male | 39 (56.5) | 53 (55.2) |  |
| Mean age at onset, years (SD) | 58.9 (12.7) | 58.9 (12.2) | 0.9791 |
| Median diagnostic delay, months (IQR) | 8.5 (5.3; 12.0) | 11.5 (7.0; 20.8) | 0.0067 |
| King’s stage at diagnosis (%) |  |  |  |
| Stage I | 7 (10.1) | 13 (13.5) | 0.8465 |
| Stage II | 31 (44.9) | 38 (39.6) |  |
| Stage III | 24 (34.8) | 33 (34.4) |  |
| Stage IV | 7 (10.1) | 12 (12.5) |  |
| Median ALSFRS-R progression per month (IQR) | -0.8 (-1.1; -0.6) | -0.7 (-1.3; -0.4) | 0.5623 |
| Median time onset to last ALSFRS-R (IQR) | 21.0 (14.0; 31.0) | 22.0 (14.0; 38.3) | 0.6766 |
| Unknown (%) | 58 (84.1) | 6 (6.3) |  |
| Median BMI loss per month (IQR) | -0.1 (-0.3; 0.0) | -0.2 (-0.4; 0.0) | 0.7095 |
| Unknown (%) | 44 (63.8) | 40 (41.7) |  |
| Therapies (%) |  |  |  |
| Riluzole | 56 (81.2) | 73 (76.0) | 0.6694 |
| Unknown | 3 (4.3) | 6 (6.3) |  |
| Percutaneous endoscopic gastrostomy | 14 (20.3) | 19 (19.8) | >0.9999 |
| Unknown | 2 (2.9) | 2 (2.1) |  |
| Invasive ventilation | 3 (4.3) | 1 (1.0) | 0.3024 |
| Unknown | 7 (10.1) | 3 (3.1) |  |
| Non-invasive ventilation | 5 (7.2) | 14 (14.6) | 0.2185 |
| Unknown | 5 (7.2) | 2 (2.1) |  |

Statistical analyses were conducted using the Fisher’s exact test, the t-test or the Mann-Whitney test. ALSFRS-R: Amyotrophic Lateral Sclerosis Functional Rating Scale Revised. BMI: Body Mass Index. GNC: General Neurological Care. IQR: Interquartile Range. SD: Standard Deviation. SMC: Specialised Multidisciplinary Care. **Supplementary table 2** Demographic and clinical characteristics of patients with bulbar onset amyotrophic lateral sclerosis, GNC cohort vs. SMC cohort.

|  | **Bulbar onset, GNC**  **n=37** | **Bulbar onset, SMC**  **n=40** | **p-value** |
| --- | --- | --- | --- |
| Sex (%) |  |  |  |
| Female | 16 (43.2) | 26 (65.0) | 0.0691 |
| Male | 21 (56.8) | 14 (35.0) |  |
| Mean age at onset, years (SD) | 62.7 (10.1) | 66.3 (8.9) | 0.0962 |
| Median diagnostic delay, months (IQR) | 9.0 (3.5; 13.5) | 9.0 (6.0; 15.0) | 0.4450 |
| King’s stage at diagnosis (%) |  |  |  |
| Stage I | 9 (24.3) | 12 (30.0) | 0.1413 |
| Stage II | 7 (18.9) | 3 (7.5) |  |
| Stage III | 19 (51.4) | 17 (42.5) |  |
| Stage IV | 2 (5.4) | 8 (20.0) |  |
| Median ALSFRS-R progression per month (IQR) | -0.6 (-1.4; -0.2) | -1.0 (-1.5; -0.8) | 0.0916 |
| Median time onset to last ALSFRS-R (IQR) | 16.0 (14.0; 67.0) | 15.5 (10.8; 27.3) | 0.2698 |
| Unknown (%) | 30 (81.1) | 6 (15.0) |  |
| Median BMI loss per month (IQR) | -0.1 (-0.4; -0.1) | -0.1 (-0.5; 0.0) | 0.5135 |
| Unknown (%) | 24 (64.9) | 18 (45.0) |  |
| Therapies (%) |  |  |  |
| Riluzole | 33 (89.2) | 32 (80.0) | 0.7106 |
| Unknown | 1 (2.7) | 3 (7.5) |  |
| Percutaneous endoscopic gastrostomy | 10 (27.0) | 16 (40.0) | 0.3350 |
| Unknown | 1 (2.7) | 0 (0.0) |  |
| Invasive ventilation | 0 (0.0) | 1 (2.5) | >0.9999 |
| Unknown | 2 (5.4) | 1 (2.5) |  |
| Non-invasive ventilation | 2 (5.4) | 5 (12.5) | 0.4378 |
| Unknown | 3 (8.1) | 1 (2.5) |  |

Statistical analyses were conducted using the Fisher’s exact test, the t-test or the Mann-Whitney test. ALSFRS-R: Amyotrophic Lateral Sclerosis Functional Rating Scale Revised. BMI: Body Mass Index. GNC: General Neurological Care. IQR: Interquartile Range. SD: Standard Deviation. SMC: Specialised Multidisciplinary Care.

**Supplementary figure 1** Overall odds ratios of medication groups (4^th^ level of the Anatomical-Therapeutic-Chemical classification) prescribed after diagnosis of amyotrophic lateral sclerosis. The odds ratios correspond to the conditional maximum likelihood estimates.

**Supplementary figure 2** Odds ratios of medication groups (4^th^ level of the Anatomical-Therapeutic-Chemical classification) prescribed in the first year after diagnosis of amyotrophic lateral sclerosis. The odds ratios correspond to the conditional maximum likelihood estimates.

**Supplementary figure 3** Odds ratios of medication groups (4^th^ level of the Anatomical-Therapeutic-Chemical classification) prescribed in the second year after diagnosis of amyotrophic lateral sclerosis. The odds ratios correspond to the conditional maximum likelihood estimates.

**Supplementary figure 4** Odds ratios of medication groups (4^th^ level of the Anatomical-Therapeutic-Chemical classification) prescribed in the third year after diagnosis of amyotrophic lateral sclerosis. The odds ratios correspond to the conditional maximum likelihood estimates.

**Supplementary figure 5** Kaplan-Meier analyses comparing survival from diagnosis between patients receiving GNC and patients receiving SMC in (A) the whole cohort (GNC 19.5 months [95% CI 15.0-25.0] vs. SMC 26.0 months [95% CI 19.0-33.0]), (B) the spinal onset ALS subgroup (GNC 16.5 months [95% CI 11.0-22.0] vs. SMC 29.0 months [95% CI 21.0-38.0]), and (C) the bulbar onset ALS subgroup (GNC 25.0 months [95% CI 18.0-29.0] vs. SMC 15.0 months [95% CI 8.0-28.0]). Median survival with the corresponding 95% CI are reported. ALS: Amyotrophic Lateral Sclerosis. GNC: General Neurological Care. SMC: Specialised Multidisciplinary Care.

**Supplementary table 3** Summary of 12-month fixed-horizon Cox regression model.

| **Variable** | **HR estimate** | **95% CI** | **p-value** |
| --- | --- | --- | --- |
| Sex [male] | 0.97 | 0.59-1.59 | 0.9082 |
| Age at onset | 1.05 | 1.02-1.08 | 0.0001 |
| King's stage II at diagnosis | 1.11 | 0.46-2.66 | 0.8178 |
| King's stage III at diagnosis | 2.29 | 1.05-5.04 | 0.0384 |
| King's stage IV at diagnosis | 2.72 | 1.07-6.93 | 0.0357 |
| Onset region [spinal] | 0.74 | 0.36-1.51 | 0.4036 |
| Care model [GNC] | 0.50 | 0.20-1.25 | 0.1375 |
| Diagnostic delay | 1.00 | 0.99-1.02 | 0.7660 |
| Onset region [spinal] : Care model [GNC] | 4.37 | 1.46-13.12 | 0.0085 |

GNC: General Neurological Care. HR: Hazard Ratio. 95% CI: 95% Confidence Interval.

**Supplementary table 4** Summary of 24-month fixed-horizon Cox regression model.

| **Variable** | **HR estimate** | **95% CI** | **p-value** |
| --- | --- | --- | --- |
| Sex [male] | 1.31 | 0.90-1.90 | 0.1526 |
| Age at onset | 1.04 | 1.02-1.06 | <0.0001 |
| King's stage II at diagnosis | 1.13 | 0.61-2.08 | 0.7010 |
| King's stage III at diagnosis | 2.08 | 1.20-3.62 | 0.0095 |
| King's stage IV at diagnosis | 2.18 | 1.08-4.40 | 0.0292 |
| Onset region [spinal] | 0.71 | 0.41-1.23 | 0.2245 |
| Care model [GNC] | 0.59 | 0.31-1.11 | 0.1029 |
| Diagnostic delay | 0.99 | 0.98-1.01 | 0.4406 |
| Onset region [spinal] : Care model [GNC] | 3.21 | 1.47-6.99 | 0.0034 |

GNC: General Neurological Care. HR: Hazard Ratio. 95% CI: 95% Confidence Interval.

**Supplementary table 5** Summary of 36-month fixed-horizon Cox regression model.

| **Variable** | **HR estimate** | **95% CI** | **p-value** |
| --- | --- | --- | --- |
| Sex [male] | 1.37 | 0.98-1.91 | 0.0648 |
| Age at onset | 1.03 | 1.01-1.05 | <0.0001 |
| King's stage II at diagnosis | 1.37 | 0.81-2.33 | 0.2406 |
| King's stage III at diagnosis | 2.07 | 1.27-3.38 | 0.0035 |
| King's stage IV at diagnosis | 1.99 | 1.04-3.80 | 0.0377 |
| Onset region [spinal] | 0.72 | 0.43-1.20 | 0.2035 |
| Care model [GNC] | 0.90 | 0.52-1.55 | 0.7043 |
| Diagnostic delay | 1.00 | 0.98-1.01 | 0.5438 |
| Onset region [spinal] : Care model [GNC] | 1.86 | 0.95-3.67 | 0.0710 |

GNC: General Neurological Care. HR: Hazard Ratio. 95% CI: 95% Confidence Interval.

**Supplementary table 6** Summary of piecewise Cox regression model, time-split at 24 months.

| **Variable*** | **HR estimate** | **95% CI** | **p-value** |
| --- | --- | --- | --- |
| Sex [male] | 1.38 | 1.02-1.88 | 0.0372 |
| Age at onset | 1.03 | 1.02-1.05 | <0.0001 |
| King's stage II at diagnosis | 1.21 | 0.77-1.92 | 0.4097 |
| King's stage III at diagnosis | 1.82 | 1.19-2.78 | 0.0057 |
| King's stage IV at diagnosis | 1.75 | 1.00-3.08 | 0.0515 |
| Onset region [spinal] | 0.62 | 0.36-1.07 | 0.0860 |
| Care model [GNC] | 0.57 | 0.30-1.07 | 0.0800 |
| Diagnostic delay | 1.00 | 0.99-1.01 | 0.9077 |
| Onset region [spinal] : Care model [GNC] (0-24 months) | 3.36 | 1.55-7.29 | 0.0021 |
| Onset region [spinal] : Care model [GNC] (24+ months) | 0.54 | 0.16-1.64 | 0.2754 |
| Onset region [spinal] : Care model [GNC] : Period [24+ months] | 0.16 | 0.04-0.61 | 0.0074 |

GNC=General Neurological Care. HR: Hazard Ratio. 95% CI: 95% Confidence Interval.
